# On Heels and Toes: How Ants Climb with Adhesive Pads and Tarsal Friction Hair Arrays

**DOI:** 10.1371/journal.pone.0141269

**Published:** 2015-11-11

**Authors:** Thomas Endlein, Walter Federle

**Affiliations:** 1 Max Planck Institute for Intelligent Systems, Heisenbergstr. 3, 70569 Stuttgart, Germany; 2 University of Cambridge, Department of Zoology, Downing Street, Cambridge, CB2 3EJ, United Kingdom; University of Zurich, SWITZERLAND

## Abstract

Ants are able to climb effortlessly on vertical and inverted smooth surfaces. When climbing, their feet touch the substrate not only with their pretarsal adhesive pads but also with dense arrays of fine hairs on the ventral side of the 3^rd^ and 4^th^ tarsal segments. To understand what role these different attachment structures play during locomotion, we analysed leg kinematics and recorded single-leg ground reaction forces in Weaver ants (*Oecophylla smaragdina*) climbing vertically on a smooth glass substrate. We found that the ants engaged different attachment structures depending on whether their feet were above or below their Centre of Mass (CoM). Legs above the CoM pulled and engaged the arolia (‘toes’), whereas legs below the CoM pushed with the 3^rd^ and 4^th^ tarsomeres (‘heels’) in surface contact. Legs above the CoM carried a significantly larger proportion of the body weight than legs below the CoM. Force measurements on individual ant tarsi showed that friction increased with normal load as a result of the bending and increasing side contact of the tarsal hairs. On a rough sandpaper substrate, the tarsal hairs generated higher friction forces in the pushing than in the pulling direction, whereas the reverse effect was found on the smooth substrate. When the tarsal hairs were pushed, buckling was observed for forces exceeding the shear forces found in climbing ants. Adhesion forces were small but not negligible, and higher on the smooth substrate. Our results indicate that the dense tarsal hair arrays produce friction forces when pressed against the substrate, and help the ants to push outwards during horizontal and vertical walking.

## Introduction

Many animals have evolved specialised adhesive pads on their feet to climb on smooth surfaces. These pads are either soft and have a relatively smooth surface (‘smooth’ pads) or they consist of a brush-like array of fine hairs (‘hairy’ pads). Adhesive pads of insects secrete small amounts of fluid between the pad and the substrate, thereby creating capillary forces.

Most animal adhesive structures are direction-dependent, i.e. they attach only when pulled towards the body but detach when pushed away from it. Such directionality has been found across a wide variety of taxa, including flies [1], bush crickets [2], ants [3, 4], cockroaches [5], tree frogs [6], spiders [7, 8], and geckos [9, 10]. While directional adhesive pads allow rapid control of attachment and detachment during locomotion, they potentially limit attachment to legs oriented in the pulling direction. For example, when climbing up a vertical surface, the hind legs point downwards and therefore need to push to balance the insect’s body weight. Are climbing insects able to produce pushing forces and to what extent are forces produced by pushing or pulling legs below or above the centre of gravity?

Many insects have different types of pad on the same leg, not only distal adhesive (‘toe’) pads but also ‘heel’ pads which are located further proximally on the tarsus. For example, beetles, cockroaches and stick insects have different types of hairy or smooth pads on their proximal tarsomeres [5, 11, 12]. It was shown for these insects that the heel pads serve other functions than the distal adhesive pad [5, 11, 13].

Beetles and cockroaches climbing upwards use the distally located adhesive pads of their front legs to pull themselves upwards, whereas the proximal pads of the hind legs are used to push. When the insects climb downwards, their pads are used in the reverse order, i.e. the front legs use the proximal pads and the hind legs the distal ones. As a result of the insects’ sprawled posture, the pushing heel pads are used when the foot is pressed against the substrate, where no adhesive forces are required. Thus, their main function is to generate friction forces [5]. How do animals climb when specialised heel pads are absent? For example, flies and some spiders only have distal adhesive pads which stick only when pulled [1, 7, 8, 14].

Ants have only one distal adhesive pad (‘arolium’) [15]. However, the ventral surface of their tarsus is covered in fine, distally pointing hairs, suggesting a possible use as attachment devices. Here, we investigate which attachment structures weaver ants use when walking or climbing. Furthermore, we study the attachment performance of the hairs on the tarsus to test whether they could produce enough friction forces to assist the animal in climbing.

## Material and Methods

### Study animals

Weaver ants (*Oecophylla smaragdina*) were kept in plastic containers in a temperature-regulated room (25–28°C on a 12:12h light/dark cycle). The ants were fed with a mixture of honey and water (1:1) as well as dead insects *ad libitum*. For the experiments, we used medium-sized ant workers (8.2 ± 2.1*mg*; *N* = 48; all values given in the text are means ± standard deviations, unless otherwise stated).

### Force measurements and video recordings of climbing ants

**Fig 1:**
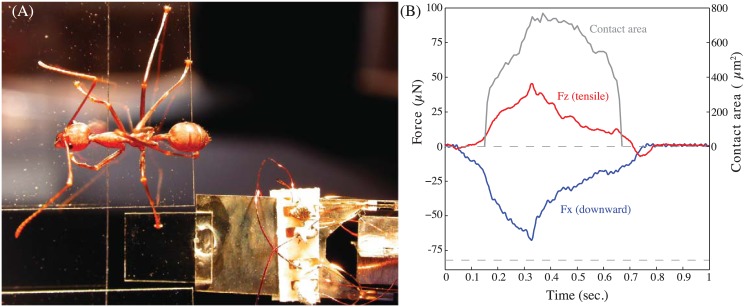
Set-up for ground reaction force recordings. (A) Weaver ants climbing on a vertical surface stepped onto a small, transparent glass plate attached to a sensitive force transducer. (B) Forces in the normal (Z) and vertical (X) directions, and contact area of the arolium for the step of a front leg above the CoM.

A custom-built, small vertical 2D force plate was used to record normal and vertical friction forces for single steps of climbing ants (Fig 1). We only considered ants that were climbing vertically upward or downward; runs with other or changing climbing directions were excluded. A piece of glass cover slip (0.1×5×5 mm) served as the walking substrate. The plate was attached to a horizontally oriented force transducer, which consisted of two M-shaped, thin metal plates (0.1 mm thickness) joined at right angle, each with four glued-on semiconductor strain gauges (Micron Instruments, USA) in full Wheatstone bridge circuit configurations. The lever arms for both directions were about 10 mm. The spring constant for both axes was ca. 17 Nm^−1^, and the resonance frequency including the glass plate ca. 80 Hz. The displacement of the substrate mounted on the force transducer for a typical step (<70 μN) was less than 5 μm, and was therefore unlikely to influence the ants’ locomotion. The data from the force transducer were recorded to a DAQ board (NI-BNC 2110, National Instruments, USA) at a frequency of 1kHz using custom Matlab scripts (Mathworks Corp., USA). The noise level for both normal and friction forces was ca. 5.6 μN.

Parallel to the force recordings, four synchronised digital high speed video cameras recorded with 100 frames per second the climbing ants when stepping on the force platform. The first camera (HotShot NAC Image Technology, USA; 1280×1024 pixels) viewed the whole ant in top view. This helped to determine the climbing direction of the ant, and to see which leg (fore, middle or hind leg) stepped onto the force plate. In addition, the direction of the leg relative to the force transducer axis could be determined.

Our 2D setup did not allow us to measure forces in the horizontal direction. However, we made the simplifying assumption that forces in the leg’s transverse direction were small and negligible (as the tarsal chain can resist larger forces only when loaded along its axis [16], whereas transverse forces would bend the chain easily). This assumption allowed us to estimate shear forces along the projected axis of the leg. We discarded recordings where the projection of the legs was oriented horizontally, i.e. approximately parallel to the transducer axis (<20° deviation), as the calculation of the force along the leg became error-prone in this range. From the dorsal view, the Centre of Mass (CoM) of the ant was approximated as the midpoint between the anterior end of the head and the posterior end of the gaster and used to determine whether a foot was placed above or below the CoM.

A second camera (Basler A602f, Germany; 656×491 pixels) viewed the underside of the glass plate in high magnification and epi-illumination in order to record the ant’s adhesive contact area during a step [4]. The third and forth camera (Basler A602f) were used in high magnification to film a single leg in side view. This allowed us to measure the angle between the tarsus and the surface three-dimensionally using the direct linear transformation method [17]. We marked the proximal and distal ends of the 5^th^ tarsomere to find its longitudinal axis. As the tarsomeres are round, tube-like structures, the ends of the tarsomeres were visible from the different camera perspectives. The 3D position of the substrate plane was measured from three marks on the surface before the recordings. This allowed us to calculate the angle between the 5^th^ tarsomere and the surface. In this paper, we refer to this angle as the ‘5^th^ tarsomere angle’. In addition to the recordings of ants climbing on a vertical surface, we filmed ants walking on horizontal and inverted glass surfaces (without force measurement).

Force and video recordings were analysed frame by frame using custom-built Matlab scripts. We measured the pad’s adhesive contact area (if present) during the stance phase, the angle of the 5^th^ tarsomere with the substrate and the ground reaction forces in the vertical and normal direction. Data were filtered using a 2^nd^ order Butterworth low-pass filter with a cut-off frequency of 20 Hz. From the smoothed data, we measured peak forces in both axes, as well as the contact area and 5^th^ tarsomere angle with the surface at the same time.

When data are presented as box-plots, the median is shown by a line, and the 25^th^ and 75^th^ percentiles by the box. The whiskers extend to the most extreme data values that are not outliers (values exceeding the 25^th^ or 75^th^ percentile by 1.5 times the interquartile range). Throughout the paper, we use means for normally distributed data, and medians otherwise. All data are available from the Dryad digital repository (http://doi.org/10.5061/dryad.vg446).

### Friction force measurements of tarsal hairs

In order to test friction forces of the hairy tarsomeres in a controlled way on different surfaces and in different directions, we used severed legs of freshly killed ants. The leg was mounted on a microscope slide such that the ventral side of the 3^rd^ and 4^th^ tarsomere formed the highest point and could be brought into contact with the surface of the force transducer.

The 2D-force transducer was moved by a motorised XZ-stage. Controlled movements were performed using a custom LabVIEW program (LabVIEW, National Instruments, USA) which allowed us to maintain a constant normal force (30 μN, 60 μN and 100 μN) during friction experiments. We measured friction forces along the proximal-distal axis of the tarsus by performing pushing and pulling slides over a distance of 300 μm at a constant speed of 60 μms^−1^. Pads were first brought into contact with the surface allowing five seconds to adjust to the preload, followed by a pull and then a push. Immediately at the end of each pushing movement, the tarsus was pulled off at an perpendicular angle with a speed of 200 μms^−1^ in order to measure adhesion.

We further tested the effect of surface roughness by using either a smooth glass cover slip (0.1×5×5 mm) or a rough polishing disc substrate (aluminium oxide, nominal particle size 1 μm, Ultratec, USA) glued onto a 5×5 mm glass coverslip. As both substrates were placed next to each other on the same force transducer, a quick and direct comparison was possible by moving the sample with help of the motorised stage between the two surfaces (visible as ‘gap’ in the time line).

The contact behaviour of the hairs against the smooth (transparent) surface was filmed by using a synchronised video camera (Basler A602f, Germany; 656×491 pixels) attached to a stereo microscope.

## Results

### Tarsus orientation during level and inverted walking

**Fig 2:**
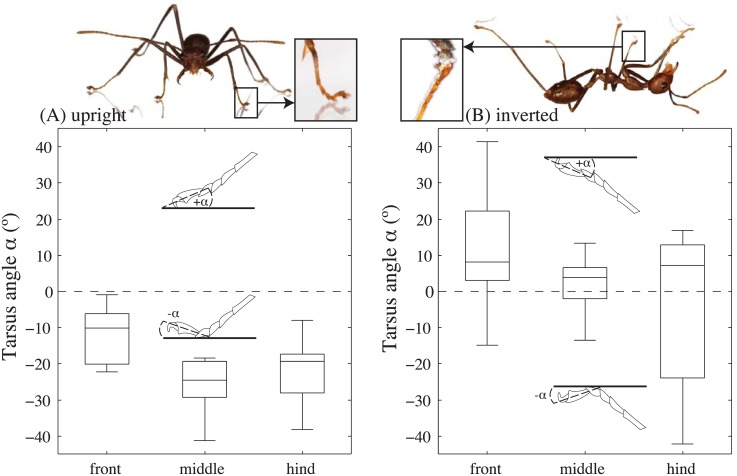
Tarsomere angle of freely walking weaver ants. (A) Level walking. Here, the ants stood mainly on their 3^rd^ and 4^th^ tarsomeres and held the 5^th^ tarsomeres away from the substrate (5^th^ tarsomere angle <0°; see photo). (B) Inverted climbing. Here, the 5^th^ tarsomere angle of was mostly positive, allowing the adhesive pad (arolium) to make contact (see also photo).

When weaver ants walked on a horizontal substrate (average speed 28.6 ± 12.5 mms^-1^;*N* = 20), the last (5^th^) tarsal segment was mostly raised so that the pretarsal claws and arolium did not touch the surface (median 5^th^ tarsomere angle -10° for front legs, -24° for the middle legs and -19° for the hind legs; Fig 2A; *N*_*front*_ = 7;*N*_*middle*_ = 10;*N*_*hind*_ = 7;). Only in 3 out of 7 cases was the 5^th^ tarsomere angle so high (>-10°) that the arolium might have touched the substrate. However, no evidence of a contact (area) was found in the recordings of the surface of the platform. This indicates that the claw flexor muscle (controlling not only the claws, but also the last tarsomere and the adhesive pad [18]) was mostly relaxed and the tarsomeres were in their raised default position.

In contrast, when ants walked on an inverted substrate (average speed 21.4 ± 14.6 mms^-1^;*N* = 21), the 5^th^ tarsomere angle was mostly positive (front legs: 8°; middle legs: 4°; hind legs: 7°; all medians; Fig 2B; *N*_*front*_ = 35;*N*_*middle*_ = 11;*N*_*hind*_ = 39) so that the adhesive pads could make contact to the substrate. In 13 out of 39 steps of hind legs the last tarsomere was again held slightly off the substrate (<0°). In these cases, ants possibly relied on their front and middle legs to adhere to the substrate.

### Ground reaction force in vertically climbing ants

#### Normal forces

**Fig 3:**
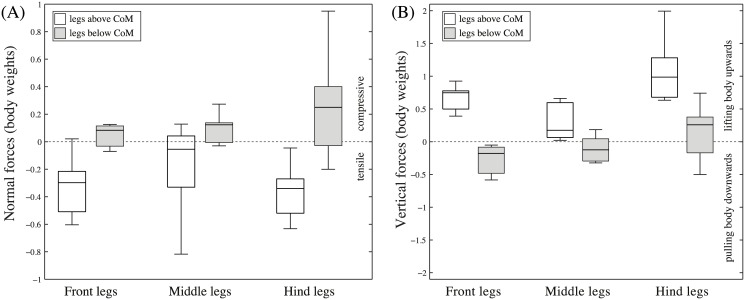
Ground reaction forces in vertically climbing weaver ants. (A) normal forces. (B) vertical forces (number of legs above (below) CoM: *N*_*front*_ = 9(3);*N*_*middle*_ = 10(8);*N*_*hind*_ = 8(18)).

When climbing on a vertical surface (average speed 21.7 ± 8.6 mms^-1^;*N* = 14), the ants’ legs produced smaller normal than shear (vertical) forces (Fig 1B). Positive normal forces were almost exclusively produced by legs below the ant’s centre of mass (CoM), helping to balance the body’s pitching moment. On average, legs above the CoM produced negative normal forces of 0.29 times body weight (Fig 3A), whereas legs below the CoM produced positive (compressive) forces of 0.12 times body weight (medians).

#### Shear (vertical) forces

The climbing ants’ legs often produced vertical force peaks exceeding the body weight, indicating that individual legs can easily support the entire body (Fig 3B). The ants held their body weight mainly by pulling with legs above the CoM (median 0.67 times body weight). Some legs below the CoM contributed to upward forces by pushing (maxima up to 0.7 times body weight), but on average their contribution to vertical forces (median 0.12 times body weight) was significantly less than that of the legs above the CoM (linear mixed-effect model; individual ants as random and leg orientation as fixed effects: *t*_9_ = −5.89, *P* < 0.001). Some legs (mainly hind legs) produced forces opposite to those of the legs above the CoM, effectively pulling the ant downward during upward climbing (negative values in Fig 3B) and upward during downward climbing (positive values in Fig 3B).

**Fig 4:**
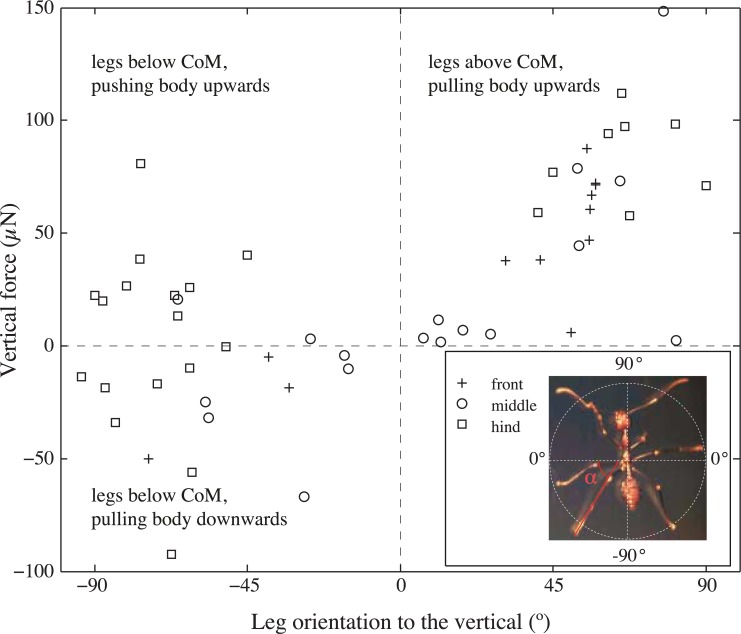
Single-leg vertical forces in weaver ants climbing on a vertical glass surface, as a function of leg orientation. Leg orientation is given as an angle ranging from -90° (pointing downward; *N*_*front*_ = 3, *N*_*middle*_ = 8, *N*_*hind*_ = 18) to 0° (horizontal) to +90° (pointing upward; *N*_*front*_ = 9, *N*_*middle*_ = 10, *N*_*hind*_ = 8). For all legs with negative angles, the foot position was below the CoM, and positive angles corresponded to foot positions above the CoM.

The same trend was also apparent when leg orientation was coded not only as above or below the CoM but more quantitatively as the leg’s angle to the vertical (Fig 4). The largest anti-gravity forces were developed by legs that were pointing upward (above the body CoM), whereas smaller anti-gravity forces or even downward pulls occurred in downward-pointing legs (below the body CoM). Vertical forces in horizontally oriented legs were generally very small, supporting our assumption that the legs’ transverse forces are negligible.

#### Adhesive pad use during climbing

**Fig 5:**
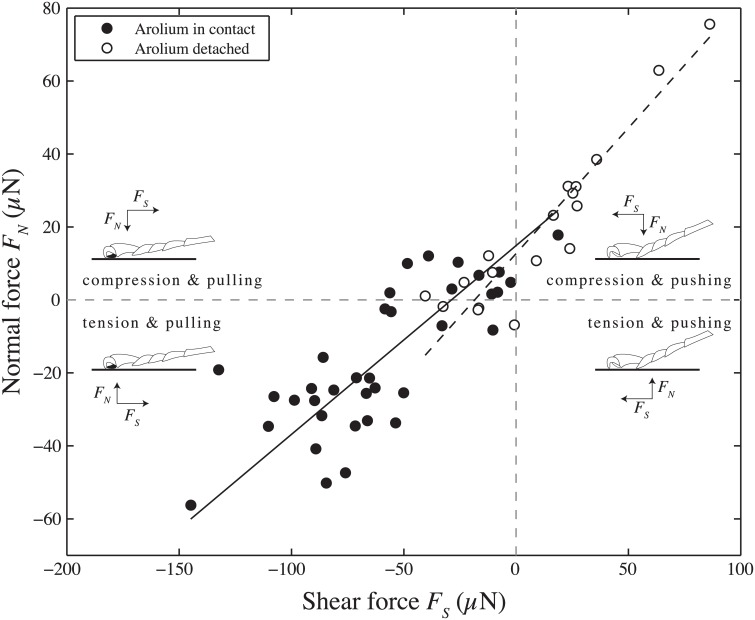
Relationship between peak normal force and corresponding shear force along the (projected) axis of the leg for individual steps. Filled circles denote steps in which the arolium made visible surface contact, whereas steps with detached arolium are marked with open circles. Lines show standardised major axis regressions on both types of steps. In most steps where the adhesive pad made contact, the legs were pulling, whereas steps without visible arolium contact occurred mostly when legs pushed. The drawings illustrate the direction of the normal forces (*F*_*N*_) and shear forces (*F*_*S*_) and the presence or absence of adhesive pad contact (black or white filling of the pad).

We compared ground reaction forces produced by legs that used the adhesive pad with those that did not. In steps with adhesive pads the normal forces were mostly negative (adhesive) and the legs pulled toward the body (lower left quadrant in Fig 5). In contrast, when the adhesive pad was not used, the legs mostly pressed onto the substrate (positive normal forces) and were used in a pushing direction (upper right quadrant in Fig 5). This is in line with previous findings that force vectors are usually aligned approximately with the legs to minimize joint torques, and that pushing leads to a detachment of the arolium [3]. Only a few cases showed legs pulling and pressing lightly into the surface (mostly with the arolium in contact, upper left quadrant in Fig 5). We never recorded any steps where the legs pushed and produced a negative normal force. Minimal adhesion during a push is probably critical for effortless and rapid detachment during locomotion, and may be a very widespread property of animal adhesive structures [19].

Both in the pushing and the pulling direction, there was an approximately linear relationship between peak normal forces and the corresponding shear forces, with a small intercept. This indicates a relatively constant angle between the force vector and the substrate. However, the angle of the force vector to the horizontal was considerably lower for ‘pulling’ steps than for ‘pushing’ ones (pulling: 7 ± 26°, pushing: 46 ± 7°; Mann-Whitney U-test: *R* = 524, *z* = 4.75, *P* < 0.001; ANCOVA to test difference between regression slopes for arolium in contact vs. detached: *F*_1,50_ = 5.88, *P* = 0.019) suggesting that the ants needed higher normal forces for producing enough friction when using their tarsal hairs. The average angle of the 5^th^ tarsomere measured from the video recordings was 12±7° for pulling legs, confirming that the tarsus approximately aligned with the force vector, whereas it was negative (i.e. raised off the substrate and not aligned) for pushing legs.

### Morphology of tarsal hairs

**Fig 6:**
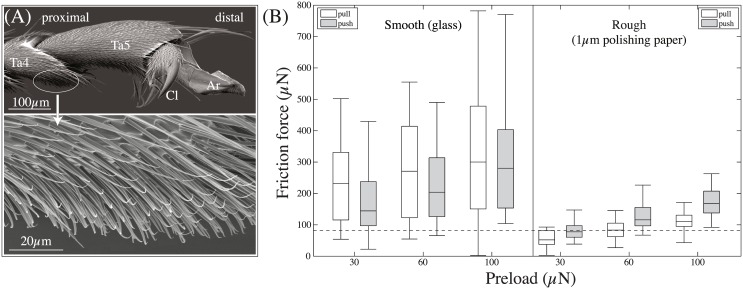
Morphology of tarsal hairs in *O. smaragdina* ants and generated friction forces. (A) Morphology of hairs on the underside of the tarsus. Ta4 & Ta5: 4^th^ & 5^th^ tarsomere, respectively; Cl: Claws; Ar: Arolium; (B) Friction forces of tarsal hairs on a smooth and a rough substrate. Slides were performed in the pulling and pushing direction with a constant normal load of 30, 60 and 100 μN. The dashed line indicates the ants’ body weight (82 μN).

When ants made steps without using the adhesive pad, the ventral side of the 4^th^ tarsomere, and sometimes of the 3^rd^ tarsomere, touched the surface. These tarsomeres are covered in fine pointed hairs that are oriented distally (Fig 6A). The density of the hairs was approx. 29466 ± 5608 hairs per mm^2^(mean ± SD of three analysed legs). Each hair was around 36±10 μm long, 2.4±0.4 μm thick at the base and tapered towards the tip (27 hairs measured from two legs of two ants). The pointed tips appear to be slightly flattened (see Fig 6A, high magnification).

### Friction and adhesion of tarsal hair arrays

We tested whether the tarsal hairs could generate enough friction on smooth and rough substrates to balance the ant’s body weight and whether the friction of tarsal hairs is direction dependent.

We found that the tarsal hairs’ friction forces were influenced by the type of surface, the direction of drag and pre-load (Repeated measures 3-way ANOVA, interaction surface × direction, *F*_1,450_ = 7.71, *P* = 0.006; load, *F*_1,450_ = 15.05, *P* < 0.001; interactions with load not significant). On the smooth surface, the friction forces generated by the tarsal hairs amounted to 3.1 times of an ant’s body weight (median), with higher friction forces in the *pulling* direction (Wilcoxon signed rank test: *R* = 1454, *z* = −5.16;*P* < 0.001). In contrast, on the rough surface, *pushing* forces were significantly higher than pulling forces (Wilcoxon signed rank test: *R* = 495, *z* = −7.87, *P* < 0.001), indicating a direction dependence caused by the interlocking of hairs with surface asperities. Both pushing and pulling forces were lower on the rough surface compared to the smooth one (Wilcoxon signed rank tests between the two surfaces for the pushing orientation: *R* = 362, *z* = −8.24, *P* < 0.001; for the pulling orientation: *R* = 45, *z* = −9.14, *P* < 0.001), possibly due to a smaller side contact area on the rough surfaces.

**Fig 7:**
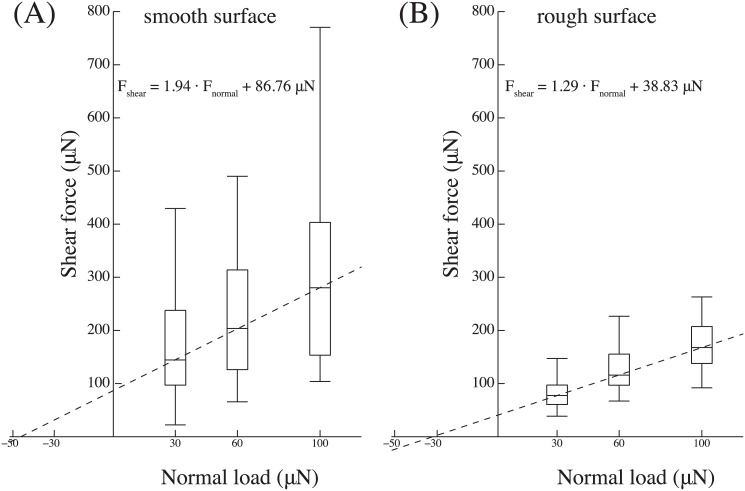
Friction forces of tarsal hairs in the pushing direction. (A) on a smooth and (B) on a rough surface for varying normal preloads (30, 60 and 100 μN). The dashed lines show the results of a linear regression on the median for each normal force level.

Friction measurements on tarsal hairs in the pushing direction with varying normal loads (30, 60 and 100 μN) showed that friction strongly increased with normal force on both smooth and rough surfaces (Fig 7, 2-way Repeated Measures ANOVA, load: *F*_1,451_ = 14.8;*P* < 0.001, surface: *F*_1,451_ = 122.9; *P* < 0.001, interaction surface × load not significant). The data suggest that the increase is approximately linear with a positive intercept (for smooth surfaces: *F*_*shear*_ = 1.94 ⋅ *F*_*normal*_ + 86.76 μN; for rough surfaces: *F*_*shear*_ = 1.29 ⋅ *F*_*normal*_ + 38.83 μN). The values correspond to a load-dependent effective friction coefficient ranging from 4.8 at 30 μN to 2.8 at 100 μN on the smooth surface and from 2.6 at 30 μN to 1.7 at 100 μN on the rough surface, respectively.

**Fig 8:**
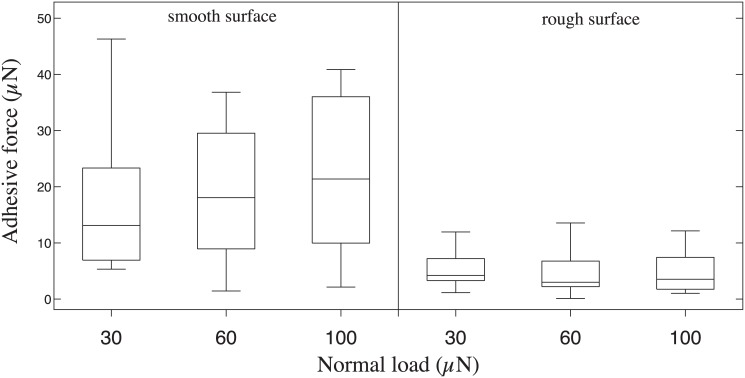
Adhesive forces of tarsal hairs on a smooth and rough surface (1 μm asperity size) after pushing slides under different normal loads (30, 60 and 100 μN).

Adhesive forces of the hair arrays were much smaller than friction forces on both substrates (Fig 8). However, adhesive force was significantly higher on the smooth surface compared to the rough surface but independent of preload (2-way ANOVA for surface *F*_1,30_ = 27.16;*P* < 0.001; preload and its interaction not significant).

**Fig 9:**
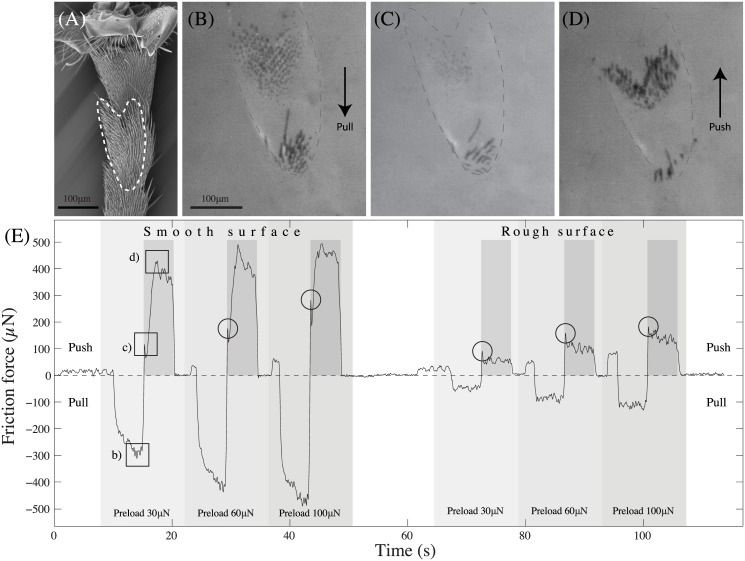
Typical friction force trace from tarsal hair fields against a smooth and rough surface. (A) SEM image showing the tarsal hairs of the 3^rd^ and 4^th^ tarsomeres (the part in contact with the surface is outlined with a dashed line). (B) Contact area of the hairs in pulling orientation using coaxial illumination on a stereo-microscope. (C) Hairs at the point of buckling. (D) Hairs re-orientated under pushing shear forces. (E) Force trace of tarsal hairs under different preloads and shearing directions on smooth and rough surfaces. Images in sub-figures (B-D) were taken at the points marked with squares in the raw trace curve. Circles mark characteristic ‘kinks’ in the force curve indicating the buckling of the hairs. Darker shaded regions indicate the time period during which the motorised stage moved the sample in the pushing direction.

The contact area recordings revealed that the hairs buckled when subjected to a high enough shear force in the pushing direction (Fig 9; see also S1 Video). When buckling, the hairs re-orientated into the proximal direction and the contact area again increased. This buckling was visible as a characteristic ‘kink’ in the friction force curve (Fig 9E, circles). These kinks allowed us to measure the hairs’ buckling forces.

While on the smooth surface, the buckling force was smaller than the maximum friction, the kink in the force trace was less well-defined on the rough surface (and no direct visualization of the contact zone was possible). Nevertheless, it is likely that buckling on the rough surface occurred at a similar time after the start of the pushing movement (Fig 9), but the forces during steady-state pushing did not rise higher than the buckling force.

The buckling forces on the smooth surface were 117±58 μN, 158±61 μN and 213±93 μN for 30, 60 and 100 μN, and 84±47 μN, 127±55 μN and 173±57 μN on the rough surface. Buckling forces were similar on smooth and rough surfaces, and significantly increased with normal force (2-way repeated measures ANOVA for load: *F*_2,37_ = 25.47, *P* < 0.001; effects of surface and interaction surface × load both not significant).

Freely walking ants produced pushing shear forces below 100 μN (maximum 86 μN; mean 32±22 μN) less than the buckling forces measured for ablated legs.

### Surface contact of tarsal hairs

**Fig 10:**
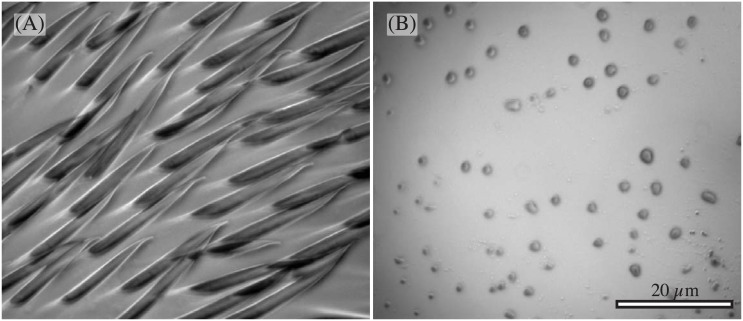
Reflected-light microscopy images of the tarsal hairs in contact with the smooth surface. (A) side contact of the hairs (B) tiny droplets left on the surface after pull-off.

As the surface contact of the tarsal hairs was often not clearly visible with the stereo microscope available during the force measurements, we used reflected-light microscopy (with a 100x oil immersion objective) to obtain higher resolution images. We observed that the ants’ tarsal hairs made side contact (Fig 10A). The contacts were up to 31.5 μm long (mean 23.8±7.7 μm) and tapered distally with an opening angle of 13.1±3.0° (*N* = 10 hairs from 2 ants). Towards the hair base (>10 μm away from the tip) the contacts were up to 3.0 μm wide (mean 2.5±0.39 μm). The total area covered was approx. 45 μm^2^.

When the tarsomeres were moved toward the surface, equivalent to an increase in normal force, the length of the side contacts increased (see S2 Video in the Supporting Information section). The length of individual hair contacts also increased when sheared in the pulling direction. Similar to adhesive pads, pulling off the tarsal hairs left behind tiny footprint droplets (Fig 10B).

## Discussion

Our results show that weaver ants use different parts of their foot in different climbing situations. When walking upside down on a smooth surface, they used only their adhesive pads. When walking upright, they mostly kept their pads off the substrate and stood on their 3^rd^ and 4^th^ tarsal segments. When climbing vertically, they engaged the adhesive pads for legs above the CoM, but mainly the 3^rd^ and 4^th^ tarsal segments for legs below the CoM. When weaver ants climbed upwards, their hind limbs produced peak anti-gravity forces of about 35% body weight. The much larger contribution to vertical forces by legs above the body CoM compared to legs below the CoM differs from previous results for cockroaches climbing on rough substrates, where forces were found to be relatively equally distributed between legs [20]. The pushing hind limbs may release constraints for the fore limbs which are in pulling orientation.

A similar division of labour between proximal and distal parts of the tarsus for walking on horizontal, inverted and vertical surfaces has been found in other insect orders (cockroaches [21], Mantophasmatodea [22] and stick insects [13]). As a result of the sprawled posture, the feet of an insect walking on the ceiling experience not only negative normal forces acting on the feet but also an inward pull. This inward pull may also be significant during vertical climbing, in particular for the two laterally oriented middle legs which produce lateral (horizontal) forces by pulling against one another. Although the lateral forces usually cancel out, this inward pull is very important as it helps to reduce the angle of the force vector with the substrate, thereby increasing adhesion as predicted by peeling theory [23, 24]. The increased adhesion via inward pulls may also explain why hind legs in ascending ants often pulled the ant *down*, even increasing the shear forces for the front legs.

Similarly, the legs of insects walking upright are not only pressing into the substrate but also pushing outward. In all insects studied, negative normal and pulling forces are resisted by distal adhesive pads, whereas pushing forces are produced by more proximal tarsal structures. For example, Clemente *et al*. [5] showed that cockroaches use only the arolium when legs are pulling, and mainly the tarsal pads (euplantulae) when pushing, and cockroach euplantulae possess anisotropic ridge structures that enhance their pushing performance on rough surfaces [25]. While stick insect euplantulae lack such a direction-dependence, their contact is strongly dependent on normal load, thereby allowing them to generate high friction only when pressed onto the substrate [13, 26]. Unlike cockroaches, mantophasmids and stick insects, ants lack specialised euplantulae, but their tarsal segments bear on the underside arrays of pointed hairs. Similar tarsal hairs are present in many other Hymenoptera [27], and these structures have thus far not been recognized as adhesive or frictional structures, nor have their properties been investigated.

Our friction experiments on single ant tarsi show that the contact of the ants’ tarsal hair arrays is strongly dependent on normal load, unlike the situation for insect adhesive pads [28, 29]. Within the range of normal loads tested, shear forces increased approximately linearly. The increase in friction forces can be explained by the tendency of the tarsal hairs to make side contact. As they lack a specialized tip structure, increasing the normal load simply brings a larger proportion of the hair’s length into surface contact. The hairs have to bend to come into contact, thereby storing elastic energy; they therefore detach easily when normal load is reduced. Consistently, the hair arrays produced relatively small adhesion, corresponding to less than half of the ant’s body weight.

The increase of shear forces with normal load may be described by a simple classic friction model that includes adhesion [30]:

$$F_{F}=\mu \left(F_{A}+F_{N}\right)$$ (1)

where *F*_*F*_, *F*_*N*_ and *F*_*A*_ are friction, normal and adhesion forces, and *μ* is the friction coefficient. On the smooth surface, a least-square linear regression on the median friction values as a function of normal load (Fig 7) gives *F*_*F*_ = 1.94 ⋅ *F*_*N*_ + 86.76 μN, suggesting an adhesive force of *F*_*A*_ = 86.76 μN/1.94 = 44.72 μN, slightly higher than the directly measured adhesion values (Fig 8). The slope of 1.94 found in this study is in the same order of magnitude as the value of 1.25 found recently for the hairy euplantulae of stick insects [26]. Thus, the hairy tarsal pads of both species produce high friction coefficients. In contrast to the euplantulae of stick insects, however, the adhesion forces found in this study were not entirely negligible. It is still unclear whether forces in this range occur during normal locomotion, or whether the ants are able to reduce them by specific detachment movements. As stick insect euplantulae are convex pads, normal load increases their projected contact area, where hairs can come into contact. By contrast, the ants’ tarsal hairs form relatively coplanar arrays, so that more hairs can come in contact simultaneously.

The relatively high friction coefficients found in our single-leg force measurements showed that the angle between the leg’s force vector and the surface can be very small (for the smooth surface *atan*(1/2.8) = 19.7°; for the rough surface *atan*(1/1.7) = 30.4°), thereby allowing the ants to push without slipping. Our ground reaction force measurements on freely climbing ants show that the force vector angles for pushing (approx. 46°) were indeed larger than these values and larger than those for pulling legs (approx. 6°). Furthermore, our analysis of the buckling forces suggests that in freely walking ants, the hair fields don’t buckle as the ant legs’ shear forces never exceeded even the smallest measured buckling force. Also, as the force vector in freely walking ants was steeper during pushing, the hairs were pressed more strongly onto the surface. This larger normal force brings a larger fraction of the hair into surface contact, effectively shortening the free base of the hair and thereby increasing the buckling force.

The ants’ tarsal hairs produced higher shear forces in the pushing direction when sliding on a rough substrate. This result may be explained by the interlocking of hair tips with surface asperities, which can only occur in the distal direction. Interlocking and axial loading may require hairs to have a relatively high bending stiffness, in conflict with their ability to make side contact. The design for tarsal friction pads may be determined by the trade-off between stiffness for interlocking and flexibility for side contacts. It is possible that for ants, pushing plays a more important role on rough substrates where adhesion can be strongly reduced, but further ground reaction force measurements on different substrates would be required to test this hypothesis.

It is possible that tarsal friction pads are less important in smaller insects (i.e. lower friction coefficients may be required), because it may be easier for them to use distal adhesive structures for pushing. The insect tarsus has a segmented, chain-like structure [31]. Pulling on this chain will align the tarsal segments along the force vector. This way the distally located pad can be used to produce friction. However, when the legs push, the tarsal chain is loaded along its axis and will tend to buckle. The buckling force for a cylinder scales with radius^4^/length^2^, i.e. with the square of its linear dimensions. Thus, the buckling stability (i.e. buckling force per body weight) of the tarsus may decrease with body mass for larger insects, assuming isometry. It may therefore be easier for small insects to produce pushing forces with distal adhesive pads, by loading the tarsus along its axis, requiring less contribution from tarsal friction pads.

Several large insects such as adult Green bush crickets (*Tettigonia viridissima*), Indian stick insects (*Carausius morosus*) and cockroaches (*Nauphoeta cinerea*), all more than 100 times heavier than Weaver ants, have additional pads (euplantulae) [2, 5, 29]. However, some similar-sized insects such as dock leaf beetles (ca. 3 times the mass of Weaver ants [29]) have been shown to produce high friction coefficients with their proximal pads. Further work is needed to test the idea that there is a correlation between the specialization of friction pads and body size.

Our study provides further evidence that the division of labour between proximal ‘friction pads’ specialised for pushing and distal ‘true’ adhesive organs is a fundamental principle that is widespread across the arthropods.

## Supporting Information

S1 Video Contact behaviour of tarsal hairs under shear. Tarsal hair fields from the 3^rd^ and 4^th^ tarsomere of severed legs were brought into contact with a smooth glass surface. With help of a motorised stage the surface was moved in the pulling and pushing direction under varying preloads. The buckling and reorientation of the hairs is visible.
(MOV)

S2 Video Surface contact of tarsal hairs under high magnification. Tarsal hair fields of severed legs were manually brought into contact with a transparent glass surface and were slowly moved up and down. The side contact of the hairs and small fluid droplets after detachment are visible.
(AVI)
